# The relationship between problematic internet use and psychological distress in older Chinese teachers (40+) during different stages of the COVID-19 pandemic: three cross-sectional studies

**DOI:** 10.3389/fpubh.2024.1442852

**Published:** 2024-12-20

**Authors:** Xiu-Mei Chen, Li-Feng Wang, Xiao-Ling Liao, Shuai Wang, Lan Yang, I-Hua Chen

**Affiliations:** ^1^School of Information Engineering, Shandong Youth University of Political Science, Jinan, China; ^2^Faculty of Education, Qufu Normal University, Qufu, China; ^3^Faculty of Education, Jiangxi Science and Technology Normal University, Nanchang, China; ^4^Faculty of Teacher Education, Dali University, Dali, Yunnan, China; ^5^Chinese Academy of Education Big Data, Qufu Normal University, Qufu, China

**Keywords:** problematic internet use, problematic smartphone use, fear of COVID-19, psychological need thwarting, burnout, mediation model, psychological distress

## Abstract

**Background:**

Despite a consensus on the negative implications of problematic internet use (PIU) for mental health, there is a notable gap in research concerning older demographics, particularly older teachers. This study aimed to investigate the prevalence of PIU and its impact on the mental wellbeing of this population.

**Methods:**

Three sub-studies were conducted: Study 1 (2020) investigated how fear induced by COVID-19 influenced PIU and psychological distress among 3,929 older teachers. Study 2 (2021) examined the effects of PIU on psychological needs thwarting (the frustration of basic psychological needs) and psychological distress, involving 3,502 participants. Study 3 (2022) explored the impact of PIU on occupational burnout and psychological distress, with 1,276 participants. The Generalized Linear Model in Jamovi 2.3.23 was used to evaluate the three mediation models.

**Results:**

The three sub-studies revealed a high prevalence of PIU among older teachers (27.4% in Study 1, 27.4% in Study 2, and 24.5% in Study 3). High levels of PIU were associated with elevated psychological distress during the 3 years of the COVID-19 pandemic. In Study 1, fear of COVID-19 indirectly influenced psychological distress through PIU as a mediator. In Studies 2 and 3, respectively, psychological needs thwarting and occupational burnout mediated the relationship between PIU and psychological distress.

**Conclusion:**

This study confirmed the high prevalence of PIU among older school teachers and the detrimental effect of PIU on psychological needs thwarting, occupational burnout, and psychological distress. Given these findings, education authorities and school management should take proactive steps to mitigate PIU and ensure the health and wellbeing of older teachers.

## 1 Introduction

The rapid proliferation of the internet, computers, smartphones, and electronic devices over recent decades has led to an increased risk of problematic internet use (PIU), a public health concern recognized across many countries by the World Health Organization (1). The COVID-19 pandemic has exacerbated this issue due to the necessity of prolonged physical isolation (2). To compensate for the lack of face-to-face social interactions, and the psychological needs unmet in their real lives, individuals increasingly rely on the internet (3–5), thereby heightening the risk of PIU (6). Numerous studies have investigated excessive internet use among adolescent students during the pandemic, revealing a worsening trend of PIU within this demographic (7–9).

PIU, an umbrella term replacing earlier terms such as internet addiction (10), internet dependence (11), and compulsive internet use (12), refers to a maladaptive pattern of internet use characterized by loss of control, negative consequences, and compulsive thoughts about internet access (13, 14). Current research on PIU includes the following two primary aspects: Firstly, studies have documented the prevalence of PIU across diverse populations as seen in Guo et al., Zhao et al., and Mohler-Kuo et al. (15–17). However, these studies have shown inconsistent results, according to Burkauskas et al. (18). This inconsistency in prevalence rates across studies complicates the understanding of PIU’s scope and impact.

Secondly, the relationship between PIU and other factors, particularly mental health, has received significant attention from researchers (19–21), with a general consensus on the negative correlation between PIU and individual mental health. Research indicates that PIU is associated with emotional disorders (22–24) and is positively correlated with stress, anxiety, and depression among university students (25–28), as well as loneliness (29, 30), low self-esteem (31, 32), and stigmatization (33). Devine et al. (34) have underscored the critical need to gather evidence of PIU from diverse individuals to achieve a comprehensive understanding of the issue. These findings highlight the importance of further research to understand the nuances of PIU and its impact on mental health across different demographic groups.

More specifically, the issue of PIU during the COVID-19 pandemic was suspected to be exacerbated due to the increased time spent online (3, 5, 18). The existing literature on PIU during the pandemic reveals several notable gaps that warrant further investigation. First, the prevalence estimates of PIU during the pandemic are highly inconsistent across studies (18). For example, Zhao et al. (16) conducted a cross-sectional study among 11,254 Chinese university students and found a PIU prevalence rate of 28.4%. In contrast, two Hungarian studies (35, 36) reported PIU prevalence rates of 3.9% (485 hospital staff members) and 5.2% (1,817 high school teachers). A Swiss study by Mohler-Kuo et al. (17) which included 1,146 children and adolescents and 1,627 young adults, estimated the PIU prevalence rate to be 30.1% for children and adolescents and 21.3% for young adults. Given this inconsistency in PIU prevalence rates, it is essential to conduct more research to better understand this issue.

Second, the majority of research on PIU has been concentrated on younger demographics, such as adolescents and university students (21, 22, 25, 26), leaving a gap in the research on PIU among older adults (40+ years) (37). This raises particular concerns regarding the increasing longevity and growing proportion of adults in the population (38). Empirical studies have not only identified the presence of PIU within this demographic (34, 38, 39), but also highlighted its potential to increase health risks, including greater social isolation, depression, and increased suicide rates (37, 39). Given the importance of improving the quality of life for older adults for familial wellbeing and social stability, especially in the context of an aging population (40, 41), it is imperative to examine the prevalence, causes, and consequences of PIU among older adults, with a particular focus on older teachers within the education system, who play a significant role and are interconnected with the student population.

Within the education system, teachers play a crucial role and have been identified as a group that experiences significant stress (42–44), especially during the COVID-19 pandemic. The pandemic led to the global closure of schools (45), significantly impacting the education sector and the teacher community (46). Teachers were confronted with the mandatory shift to online teaching (47) and faced technical and logistical challenges as they transitioned from classroom to home-based instruction (48), along with increased workloads and diminished communication with students and parents (49). Consequently, the literature has shown that COVID-19 has inflicted considerable psychological distress and excessive internet use on teachers (50, 51). Some studies explored the impact of teachers’ PIU on their mental health (44, 52, 53).

Addressing the prevalence and impact of PIU among teachers is essential, as their psychological wellbeing greatly affects teaching effectiveness (54). Previous research has shown that teachers’ mental health is closely related to student behavior, potentially leading to worse academic achievement and reduced student motivation (55, 56). While these studies have shed light on the impact of teachers’ PIU on their mental health, a significant gap remains in the literature, as there has been no specific focus on older teachers. This oversight is a critical area for future research, as understanding the challenges faced by older teachers can inform targeted interventions and support strategies to promote healthy internet use and overall wellbeing.

Third, most current studies report the prevalence of PIU among participants using a single cross-sectional design. While this approach captures the relationship between PIU and individual psychological distress at a specific moment, it falls short of providing a comprehensive analysis. A more robust methodology would be to employ multiple cross-sectional studies (called repeated cross-sectional studies) conducted over time, which would offer a dynamic view of how PIU prevalence has fluctuated among target groups and how it has interacted with psychological distress throughout different phases of the COVID-19 pandemic.

In a prior study, Soriano et al. utilized a repeated cross-sectional survey to study the prevalence of chronic obstructive pulmonary disease (COPD) in Spain using two different samples at two distinct points in time: 1997 and 2007 (57). Similarly, to ascertain the prevalence of PIU among older school teachers during the 3 years of the COVID-19 pandemic, we conducted a repeated cross-sectional survey at three different periods. This methodology allows researchers to gain a deeper understanding of how the population engaging in PIU has changed over time and the association between PIU and mental health outcomes as the pandemic progressed and societal circumstances changed.

To address these gaps, this study focuses on older teachers in China, conducting three cross-sectional studies to evaluate their prevalence of PIU across 3 years of the COVID-19 pandemic (2020, 2021, and 2022) and examining the impact of PIU on psychological distress. The first study, set in the immediate aftermath of the pandemic outbreak (August to November, 2020), explores the influence of pandemic fear on PIU and psychological distress among older teachers. This study aims to understand how the initial shock and uncertainty of the pandemic affected teachers’ problematic online behaviors and mental wellbeing. The second study, conducted during the mid-pandemic period of online teaching in schools (November, 2021), investigates the impact of PIU on teachers’ psychological need thwarting (PNT) related to online teaching and distress. This study examines how the shift to remote education and the increased reliance on technology may have exacerbated PIU and its negative consequences for teachers’ psychological needs and wellbeing. The third study, carried out during the late-pandemic transition back to offline teaching (January, 2022), examines the effects of PIU on occupational burnout and psychological distress among older teachers. This study aims to understand how the lingering effects of PIU, developed during the pandemic, may continue to impact teachers’ professional and mental health as they readjust to in-person teaching.

In this study, we employ problematic smartphone use (PSU) as an indicator of PIU among older teachers. This decision is based on the widespread use of smartphones in China, with 99.9% of Chinese internet users accessing the internet via mobile phones, and older individuals (aged 40 and above) accounting for a significant 48.5% of this population. Moreover, there is a significant overlap between PSU and PIU (58), and a growing body of research demonstrates the close relationship between PSU and mental health (59, 60).

By conducting these three sub-studies, this research provides a comprehensive analysis of PIU and its effects on older teachers’ psychological distress throughout the different stages of the COVID-19 pandemic. The following sections will detail the research hypotheses for each sub-study.

## 2 Research hypothesis

**Figure 1:**
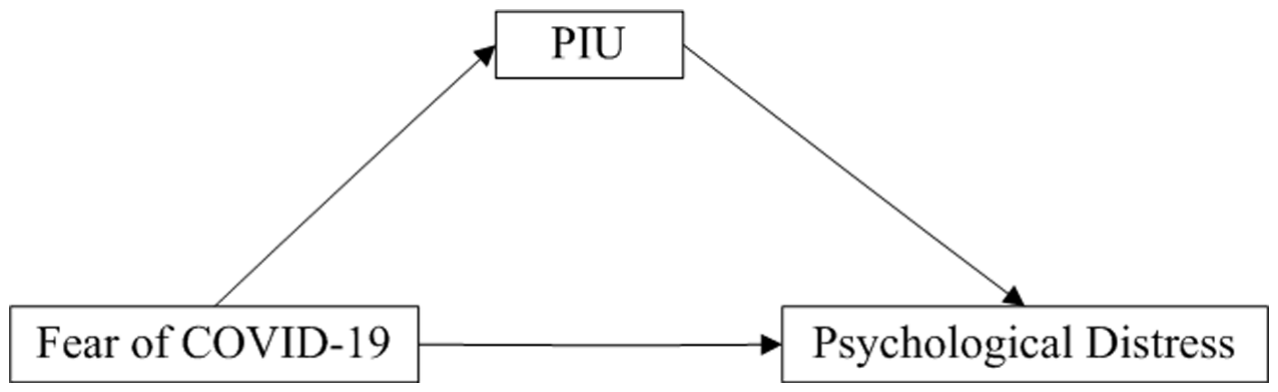
The conceptual framework of Study 1. PIU, problematic internet use.

**Figure 2:**
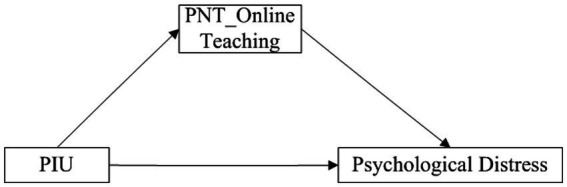
The conceptual framework of Study 2. PIU, problematic internet use; PNT, psychological need thwarting.

**Figure 3:**
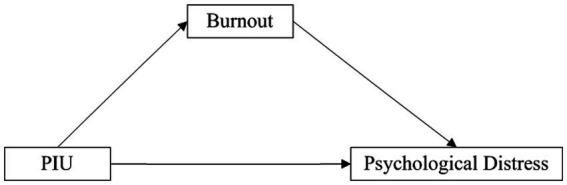
The conceptual framework of Study 3. PIU, problematic internet use.

Three conceptual models were tested in three distinct studies, and their respective hypotheses are depicted in Figures 1–3.

### 2.1 Hypotheses for study 1: the effect of fear of COVID-19 on problematic internet use, and psychological distress

The COVID-19 pandemic has led to widespread fear and the implementation of preventive measures like physical distancing. As a result, people have increasingly turned to social networks for medical information, stress relief, and psychological comfort, potentially leading to PIU (61, 62). Teachers face additional challenges that may exacerbate PIU, such as the need to use social networks for online teaching and communication with students and parents (48). Thus, we hypothesize that fear of COVID-19 is significantly and positively correlated with PIU among older teachers.

The widespread fear caused by the COVID-19 pandemic has not only been associated with increased PIU but also with various forms of psychological distress, including depression, anxiety, and post-traumatic stress (44, 63–65). Specifically, Yi et al. (44) found that Chinese primary and middle school teachers’ fear of COVID-19 was closely related to their psychological distress. As older teachers are a subset of this group, we hypothesize that their fear of COVID-19 is positively correlated with psychological distress.

Moreover, according to the Resource Model of Self-Control (66), PIU consumes a significant amount of an individual’s limited self-control resources (67). For older teachers, who have relatively less energy, this may lead to ego depletion (66) and ultimately result in psychological distress (68). Research has shown that PIU is significantly associated with the psychological wellbeing of primary and middle school teachers (44, 52, 53). Therefore, we hypothesize that PIU is positively correlated with psychological distress among older teachers.

Considering the relationships between fear of COVID-19, PIU, and psychological distress, we speculate that PIU may have a mediating effect between fear of COVID-19 and psychological distress. In other words, fear of COVID-19 may influence psychological distress through increased PIU. Thus, we hypothesize that PIU mediates the relationship between fear of COVID-19 and psychological distress.

### 2.2 Hypotheses for study 2: the effect of problematic internet use on psychological need thwarting of online teaching and psychological distress

Drawing from the Resource Model of Self-Control, PIU leads teachers to invest significant time and energy in online activities, consuming their self-control resources and resulting in ego depletion (66), which in turn affects their psychological distress (refer to the above hypothesis in study 1).

Moreover, during the COVID-19 pandemic, teachers have faced numerous challenges in adapting to online teaching, which has led to increased stress and workload (44, 68). When teachers engage in PIU, it can further exacerbate these challenges by taking time and attention away from important professional duties, such as preparing and delivering online lessons, providing feedback on student work, and maintaining communication with colleagues, students, and parents. This reduced engagement in core responsibilities can contribute to lower job satisfaction and a sense of burnout among teachers. Therefore, the combination of pandemic-related stressors and PIU may thwart teachers’ basic psychological needs for autonomy, competence, and relatedness, as they struggle to effectively manage their time, utilize new technologies, and maintain meaningful interactions with others in their professional lives.

According to self-determination theory (SDT), when these three basic psychological needs are not met, individuals experience frustration, leading to PNT (69). PNT encompasses the thwarting of autonomy, competence, and relatedness needs (69, 70). For older teachers, their lack of proficiency in technology use and unfamiliarity with online teaching would increase the frustration with their own competence (i.e., competence thwarting) (48, 71). In fact, teachers had to conduct online teaching following the rules enforced by the education sector, which may have negatively impacted teachers’ sense of autonomy (i.e., autonomy thwarting) (72). And the low teaching motivation and isolation due to online classes, suggest that they experience relatedness thwarting (53). Previous research has shown that PIU is positively correlated with PNT among primary and middle school teachers (44). Thus, we hypothesize that PIU is significantly positively correlated with PNT among older teachers.

Existing research has demonstrated that PNT in online teaching is significantly positively correlated with psychological distress among primary and middle school teachers (44, 53). Moreover, Yi et al. (44) found that PIU had an indirect positive effect on psychological distress through the mediator of PNT in online teaching. Based on this, we hypothesize that among older teachers, PIU influences psychological distress through the mediator of PNT in online teaching, implying that PNT has a mediating effect between PIU and psychological distress.

### 2.3 Hypotheses for study 3: the effect of problematic internet use on burnout and psychological distress

As discussed in Study 1, PIU impacts the psychological distress of older teachers. Moreover, according to the Resource Model of Self-Control, PIU leads to teachers’ ego depletion and emotional exhaustion (66). Exhaustion, as a core dimension of burnout (73), has been shown to be associated with students’ PIU in numerous empirical studies (74, 75). The positive relationship between teachers’ PIU and occupational burnout has also been demonstrated in existing research (52), with results indicating that PIU is significantly correlated with burnout among Hungarian high school teachers. Furthermore, a substantial body of literature has shown that burnout is closely related to psychological distress (76, 77). Therefore, we postulate that in the late stage of the COVID-19 pandemic in China, PIU leads to occupational burnout among older teachers, ultimately impacting their mental health. Based on this, we further hypothesize that occupational burnout mediates the relationship between PIU and psychological distress.

## 3 Study 1

### 3.1 Method

#### 3.1.1 Participants and procedure

The pandemic outbreak resulted in the closure of educational institutions and a subsequent transition to online teaching modalities. During the critical period from August to November 2020, Study 1 was conducted utilizing convenience sampling, a method selected due to its feasibility in accessing the target population. Ethical approval for this study was obtained from the Institutional Review Board of the Jiangxi Psychological Counselors Association (IRB: JXSXL-2020-J013). The Jiangxi and Sichuan Education Bureaus assisted in data collection by inquiring about teachers’ willingness to participate. The online survey platform was designed to require completion of all questions before submission, ensuring no missing data in the final dataset. To ensure the quality and validity of the data, we implemented stringent criteria for participant inclusion. Initially, we selected participants aged between 40 and 60 years, followed by the exclusion of those with completion times of less than 120 s, which were considered inadequate to guarantee serious engagement with the survey content. After applying these criteria, from an initial pool of 9,030 participants, the refined dataset comprised 3,929 participants (29.7% male, 70.3% female; age range: 40–60 years). Supplementary Table S1 provides detailed demographic information.

#### 3.1.2 Measures

Study 1 used three scales to measure the variables: the Fear of COVID-19 Scale (FCV-19S), Smartphone Application-Based Addiction Scale (SABAS), and the 21-item Depression, Anxiety, and Stress Scale (DASS-21). All scale items are listed in Supplementary Table S2. The measures are described below.

The FCV-19S (78) is a 7-item measure that assesses an individual’s fear of COVID-19 using a 5-point Likert scale ranging from 1 (*“Totally disagree”*) to 5 (*“Completely agree”*). Higher scores on the FCV-19S indicate greater levels of fear. The Chinese version of the FCV-19S has been validated by Chen et al. (79) on a sample of primary and middle school teachers. The Chinese version of FCV-19S has demonstrated a unidimensional structure and strong psychometric properties (79). In the current study, the scale demonstrated good internal consistency reliability, with a Cronbach’s alpha coefficient of 0.895 and a McDonald’s omega coefficient of 0.896. Sample items include: *“It makes me uncomfortable to think about Coronavirus-19.”*

PSU, as an indicator of PIU, was measured using the 6-item SABAS (80), which employs a 6-point Likert scale ranging from 1 (*“Strongly disagree”*) to 6 (*“Strongly agree”*). Higher scores indicate more PIU. The Chinese version of the SABAS has demonstrated a unidimensional structure and strong psychometric properties (81, 82). In the present study, it demonstrated satisfactory internal consistency (Cronbach’s *α* = 0.877; McDonald’s *ω* = 0.880). Sample items include: *“My smartphone is the most important thing in my life.”*

Psychological distress was assessed using the Chinese version of the DASS-21 (83, 84). The DASS-21 uses a 4-point scale ranging from 0 (*“Never”*) to 3 (*“Almost always”*), with higher scores indicating more severe psychological distress. The scale has satisfactory psychometric properties and is considered a valid indicator of general psychological distress (85, 86). In the current study, the internal consistency of the DASS-21 was excellent (Cronbach’s *α* = 0.968; McDonald’s *ω* = 0.968). Sample items include: *“I could not seem to experience any positive feeling at all.”*

#### 3.1.3 Data analysis

Descriptive statistics were calculated for each variable. Recommended cutoff values were used to examine the severity of fear of COVID-19 [cutoff = 17.5; (87)], PSU [cutoff = 21; (88)], and psychological distress [cutoff = 34; (89)] among older teachers. Pearson correlation analysis was used to analyze the relationships between the variables by using SPSS 25. The Generalized Linear Model (GLM) in Jamovi 2.3.23 was employed to test the mediation effect, with psychological distress as the dependent variable, fear of COVID-19 as the independent variable, and PIU as the mediator, controlling for age and gender.

### 3.2 Results

The prevalence rates of fear of COVID-19, PIU, and psychological distress among the 3,929 older teachers in Study 1 were 28.7, 27.4, and 2.8%, respectively (see Supplementary Table S3). These rates represent the percentage of participants surpassing the established cutoffs for abnormal levels of fear of COVID-19, PIU, and psychological distress. All three variables were significantly and positively correlated, with coefficients ranging from 0.35 to 0.45, as detailed in Supplementary Table S3. Notably, the strongest correlation was observed between fear of COVID-19 and psychological distress, with a coefficient of 0.45 (*p* < 0.01).

**Figure 4:**
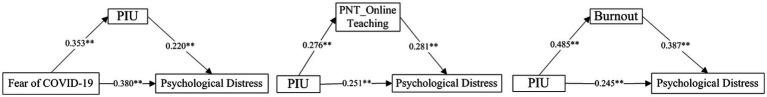
The results of the mediation analysis in the three sub-studies. PIU, problematic internet use.

The mediation analysis (see Table 1 and Figure 4) showed that fear of COVID-19 was significantly associated with both PIU (*β* = 0.353, 95% CI [0.486, 0.574], *p* < 0.001) and psychological distress (*β* = 0.380, 95% CI [1.650, 1.923], *p* < 0.001). PIU was also significantly associated with psychological distress (*β* = 0.220, 95% CI [0.598, 0.780], *p* < 0.001), after controlling for gender and age. The bias-corrected bootstrapping mediation test confirmed the significant indirect effect of fear of COVID-19 on psychological distress through PIU (95% CI [0.309, 0.422]).

**Table 1:** Results of mediation analysis (coefficient and bootstraps confidence interval) of Study 1.

	Mediator model					Dependent model				
	B (se)	*t*	95% CI		*β*	B (se)	*t*	95% CI		*β*
			LLCI	ULCI				LLCI	ULCI	
Sex (Ref: Male)	0.173 (0.206)	0.839	−0.231	0.578	0.013	−4.339 (0.598)	−7.257	−5.511	−3.166	−0.101
Age	−0.023 (0.035)	−0.660	−0.093	0.046	−0.010	0.067 (0.102)	0.654	−0.134	0.268	0.009
Fear of COVID 19	0.530 (0.023)	23.578	0.486	0.574	0.353	1.787 (0.070)	25.687	1.650	1.923	0.380
PIU						0.689 (0.046)	14.904	0.598	0.780	0.220

PIU, problematic internet use.

## 4 Study 2

### 4.1 Method

#### 4.1.1 Participants and procedure

Study 2 was conducted in November 2021 during the strict COVID-19 lockdown in China, where new cases led to local lockdown and a shift to online teaching. The online survey, approved by the Institutional Review Board of the Jiangxi Psychological Counselors Association (IRB: JXSXL-2020-J013), was voluntarily completed by teachers. The local education bureau assisted in data collection via hyperlinks to 9,242 teachers by convenience sampling. Following the inclusion of participants aged between 40 and 60 years, and then the exclusion of those with response times under 120 s, the dataset encompassed 3,502 primary and middle school teachers from a city in Jiangxi Province. The gender composition was nearly equal, with 50.1% male and 49.9% female. Supplementary Table S4 presents detailed demographic information.

#### 4.1.2 Measures

As described in Study 1, the SABAS was used to measure participants’ excessive smartphone use. The internal consistency of SABAS was satisfactory in Study 2 (Cronbach’s *α* = 0.849; McDonald’s *ω* = 0.854).

The Psychological Need Thwarting Scale of Online Teaching (PNTSOT) (44) was used to assess older schoolteachers’ PNT toward online teaching during the period of school closure. The PNTSOT comprises three subscales (i.e., autonomy, competence, and relatedness thwarting) and is rated on a seven-point Likert-type scale, with responses ranging from 1 (*“Strongly disagree”*) to 7 (*“Strongly agree”*) (70). Higher scores indicate more serious PNT during online teaching tasks. A sample item from the scale is *“In online courses during the pandemic, I cannot decide for myself how I want to teach.”* For a comprehensive list of items in the PNTSOT, refer to Supplementary Table S2. Yi et al. (44) found sound factorial validity of the scale among schoolteachers, and the internal reliability of the PNTSOT was very good in the present study (Cronbach’s *α* = 0.867; McDonald’s *ω* = 0.869). Following Yi et al. (44), the three kinds of PNT were treated as an overall construct.

The DASS-21 was used to measure psychological distress, as described in Study 1. In Study 2, Cronbach’s *α* = 0.865 and McDonald’s *ω* = 0.889.

#### 4.1.3 Data analysis

Following the data analysis outlined in Study 1, we conducted descriptive analyses, correlation analysis, and mediation analysis using the GLM in Jamovi 2.3.23. In Study 2, psychological distress served as the dependent variable, PIU as the independent variable, and PNT as the mediator. We controlled for age and gender as potential confounding variables.

### 4.2 Results

In Study 2, the prevalence rates of PIU and psychological distress among the 3,502 older teachers were 27.4 and 3.9%, respectively (Supplementary Table S5). The three variables (PIU, PNT, and psychological distress) were significantly and positively correlated, with coefficients ranging from 0.28 to 0.35, as presented in Supplementary Table S5. The most robust correlation was identified between PNT and psychological distress, with a coefficient of 0.35 (*p* < 0.01).

The results of the mediation analysis (see Table 2 and Figure 4) showed that PIU was significantly associated with both PNT (*β* = 0.276, 95% CI [0.462, 0.581], *p* < 0.001) and psychological distress (*β* = 0.251, 95% CI [0.780, 1.001], *p* < 0.001) among older teachers. Additionally, PNT showed a significant positive association with psychological distress (*β* = 0.281, 95% CI [0.467, 0.585], *p* < 0.001). A bias-corrected bootstrapping mediation test confirmed that the impact of PIU on older teachers’ psychological distress, mediated by PNT, was statistically significant (95% CI [0.230, 0.318]).

**Table 2:** Results of mediation analysis (coefficient and bootstraps confidence interval) of Study 2.

	Mediator model					Dependent model				
	B (se)	*t*	95% CI		*β*	B (se)	*t*	95% CI		*β*
			LLCI	ULCI				LLCI	ULCI	
Sex (Ref: Male)	−2.100 (0.368)	−5.71	−2.821	−1.378	−0.095	−0.701 (0.659)	1.06	−1.993	0.592	−0.017
Age	0.185 (0.036)	5.16	0.114	0.255	0.086	0.004 (0.064)	0.07	−0.121	0.130	0.001
PIU	0.522 (0.030)	17.17	0.462	0.581	0.276	0.890 (0.056)	15.78	0.780	1.001	0.251
PNT_Online Teaching						0.526 (0.030)	17.44	0.467	0.585	0.281

PIU, problematic internet use; PNT, psychological need thwarting.

## 5 Study 3

### 5.1 Method

#### 5.1.1 Participants and procedure

As the COVID-19 situation improved, Jiangxi Province resumed offline teaching in January 2022, and Study 3 was conducted during this period. The online survey for Study 3 was approved by the Institutional Review Board of the Jiangxi Psychological Counselors Association (IRB: JXSXL-2020-J013), and the local education bureau assisted in collecting online survey data through hyperlinks. Participation was voluntary, and a total of 4,045 primary and secondary school teachers took part in the survey. The online survey platform required all questions to be answered before submission, resulting in no missing data in the final dataset. After applying the inclusion criterion of participants aged between 40 and 60 years and then excluding those with response times less than 120 s, the final dataset comprised 1,276 individuals, with a gender distribution of 49.5% males and 50.5% females. Supplementary Table S6 presents detailed demographic information of the participants, providing insights into the composition of the older teachers group.

#### 5.1.2 Measures

As in the previous studies, the SABAS was used to measure the level of PIU among older teachers. The internal consistency of SABAS in Study 3 was satisfactory (Cronbach’s *α* = 0.878; McDonald’s *ω* = 0.883).

Study 3 assessed older school teachers’ occupational burnout using the “Emotional Exhaustion” subscale (eight items) of the Chinese version of the Primary and Secondary School Teachers’ Job Burnout Questionnaire (CTJBQ) (90). The CTJBQ was developed with reference to the MBI structure (91) to accommodate the cultural and linguistic background of mainland Chinese teachers. Emotional exhaustion was chosen as it contributes most significantly to burnout (91, 92). Participants rated the frequency of their experiences on a 7-point Likert scale, ranging from 1 (*“Never”*) to 7 (*“Daily”*). An example item is *“I feel that teaching is genuinely an exhausting job for me.”* Detailed items of this scale are provided in Supplementary Table S2. Higher scores indicated greater occupational burnout. Internal consistency for burnout scores was good (Cronbach’s *α* = 0.950; McDonald’s *ω* = 0.951).

The DASS-21 was used to measure psychological distress, as described in Study 1 and Study 2. In Study 3, Cronbach’s *α* = 0.967 and McDonald’s *ω* = 0.967.

#### 5.1.3 Data analysis

Consistent with the methodologies employed in the preceding studies, Study 3 involved a series of statistical analyses, including descriptive analyses, correlation analysis, and mediation analysis using the GLM in Jamovi 2.3.23. In this analysis, we examined occupational burnout as a mediator in the relationship between PIU and psychological distress, controlling for age and gender.

### 5.2 Results

Study 3 found the prevalence rates of PIU and psychological distress among the 1,276 older teachers to be 24.5 and 5.6%, respectively (Supplementary Table S7). PIU, occupational burnout, and psychological distress were significantly and positively correlated, with coefficients ranging from 0.43 to 0.51, as presented in Supplementary Table S7. The strongest correlation was observed between occupational burnout and psychological distress, with a correlation coefficient of 0.51 (*p* < 0.01).

The mediation analysis (see Table 3 and Figure 4) showed significant positive associations between PIU and occupational burnout (*β* = 0.485, 95% CI [0.846, 1.032], *p* < 0.001) and between PIU and psychological distress (*β* = 0.245, 95% CI [0.740, 1.145], *p* < 0.001). PIU was also significantly associated with psychological distress (*β* = 0.387, 95% CI [0.666, 0.875], *p* < 0.001). The bias-corrected bootstrapping mediation test confirmed the significant indirect effect of PIU on psychological distress through occupational burnout (95% CI [0.602, 0.845]).

**Table 3:** Results of mediation analysis (coefficient and bootstraps confidence interval) of Study 3.

	Mediator model					Dependent model				
	B (se)	*t*	95% CI		*β*	B (se)	*t*	95% CI		*β*
			LLCI	ULCI				LLCI	ULCI	
Sex (Ref: Male)	−0.258 (0.608)	−0.424	−1.450	0.935	−0.011	0.209 (1.157)	0.18	−2.061	2.479	0.004
Age	−0.016 (0.060)	−0.260	−0.134	0.102	−0.007	−0.072 (0.115)	−0.632	−0.297	0.152	−0.015
PIU	0.939 (0.048)	19.78	0.846	1.032	0.485	0.943 (0.103)	9.12	0.740	1.145	0.245
Occupational burnout						0.771 (0.053)	14.44	0.666	0.875	0.387

PIU, problematic internet use.

## 6 Discussion

Our three-year investigation in China, comprising three distinct cross-sectional studies, examined the relationship between PIU and mental health among older teachers during the COVID-19 pandemic. Using a threshold of 21 points to identify PIU (89), we found consistently high prevalence rates across the three-year period: 27.4, 27.4, and 24.5%, respectively. These findings suggest persistent PIU concerns among older teachers, though it’s important to note these rates come from different participant cohorts.

Comparing our findings with other studies on PIU during the pandemic reveals both similarities and differences. Our results align with Zhao et al. (16), who reported a PIU prevalence of 28.4% among Chinese university students. Mohler-Kuo et al. (17) found a slightly lower prevalence of 21.3% among Swiss young adults. However, two Hungarian studies (35, 36) reported significantly lower PIU prevalence rates, below 6%. These variations highlight the importance of context-specific analyses in understanding PIU patterns during the pandemic.

The cultural context of our study may also play a role in the prevalence of PIU. In Asian countries, cultural norms might limit opportunities for free interaction and self-expression (93), leading to increased reliance on mobile devices. This cultural backdrop could explain the high PIU prevalence observed in our study, as individuals may have used the internet to cope with pandemic-induced stress and isolation, particularly among older adults. This insight underscores the importance of considering cultural factors when examining internet usage behaviors during crises.

The compensatory internet use theory (6) offers a valuable perspective for understanding the connection between fear of COVID-19 and PIU. It suggests that individuals may turn to the internet as a way to fulfill psychological needs that are not being met in their offline lives. Consequently, the stress and isolation exacerbated by the pandemic could potentially drive individuals to seek solace in internet engagement as a means to mitigate their fears regarding COVID-19.

Furthermore, our study consistently demonstrated the significant relationship between PIU and psychological distress among older teachers, even after controlling for gender and age. These results align with previous research that has established the negative impact of PIU on mental health (22–27), including anxiety, depression and suicidal thoughts. The Resource Model of Self-Control (66) provides a theoretical framework to understand this relationship, suggesting that PIU depletes individuals’ limited self-control resources, leading to ego depletion and ultimately resulting in psychological distress (73).

Building upon the direct effect of PIU on psychological distress, this study also revealed the mediating roles of PNT and occupational burnout in the relationship between PIU and psychological distress. These findings are consistent with self-determination theory (69) and previous research on the relationship between PIU and burnout (53) and the association between burnout and psychological distress (76, 77). The thwarting of basic psychological needs for autonomy, competence, and relatedness in online teaching, as well as the experience of occupational burnout during the transition back to offline teaching, serve as important mechanisms through which PIU contributes to psychological distress among older teachers.

Notably, despite different participants, the impact coefficient of PIU on the psychological distress of older teachers generally showed a stable trend, with *β* values ranging from 0.220 (Study 1), 0.251 (Study 2), to 0.245 (Study 3). This demonstrated that the harm caused by PIU to the mental health of this population remained consistent, suggesting that for older teachers, the adverse impact of PIU on psychological distress is persistent, and should draw the attention of relevant educational departments.

Our findings suggest a complex interplay of factors associated with PNT and occupational burnout among educators during different phases of the pandemic. Beyond our primary variables of interest, research indicates several other important factors in understanding teacher wellbeing. Bartholomew et al.’s study (94) showed associations between job pressure and the thwarting of autonomy, competence, and relatedness needs among physical education teachers. Chen et al. (95) found correlations between teachers’ perceived instructional leadership and burnout levels. School environment has also been linked to teacher burnout patterns (96). These findings indicate that the relationships between PIU, PNT, and burnout exist within a broader context of organizational and environmental factors. Understanding these multifaceted relationships could be particularly relevant when examining teachers’ experiences across different pandemic phases—from initial adjustment to online teaching through the return to in-person instruction. Future research would benefit from examining this broader range of variables to provide a more comprehensive understanding of teachers’ psychological wellbeing during periods of educational transition.

## 7 Implications

The findings of this study have important implications for the wellbeing of older teachers in China and beyond. Given the significant impact of PIU on psychological distress, it is crucial to develop and implement targeted interventions to promote healthy smartphone use and mitigate the negative effects of PSU on mental health. These interventions should not only focus on reducing excessive smartphone use but also address the underlying psychological factors that contribute to psychological distress, such as the thwarting of basic psychological needs and the experience of occupational burnout. Additionally, interventions should be sensitive to the cultural context in which they are implemented, taking into account the unique challenges and pressures faced by teachers in different settings.

In light of these considerations, we propose the following strategic directions. Firstly, educational institutions and policymakers should consider integrating digital literacy and mental health education into professional development programs for teachers. This would equip them with the skills to navigate the digital landscape responsibly and maintain a healthy balance between professional and personal smartphone use. Secondly, there is a need for the establishment of support systems within schools and educational communities, such as counseling services and peer support groups, to address the psychological distress experienced by teachers. These systems should be designed to identify and mitigate the factors contributing to occupational burnout and the thwarting of psychological needs. Lastly, policymakers should advocate for research-informed policies that promote a healthy work-life balance for educators, including guidelines on reasonable working hours and the responsible use of digital technologies in educational settings.

## 8 Strength and limitation

This study offers several notable methodological contributions. The repeated cross-sectional design captured data across three distinct phases of the COVID-19 pandemic, providing snapshots of PIU and psychological wellbeing among older teachers over time. The geographically diverse sample from various regions in China allowed for examination of PIU patterns across different educational contexts. Additionally, the use of validated measurement instruments with demonstrated reliability, combined with substantial sample sizes at each time point, strengthens confidence in the observed relationships between PIU, psychological need thwarting, occupational burnout, and psychological distress.

Despite these strengths, the study has several limitations that should be acknowledged. First, despite employing a repeated cross-sectional survey methodology, the absence of a longitudinal design constrains our capacity to delve into the developmental trajectories of the PIU prevalence and its psychological distress implications. Additionally, the cross-sectional nature of our research precludes the drawing of causal inferences, thus necessitating a cautious consideration of the potential for reverse causality. To address these limitations and to elucidate the causal pathways, future research should adopt a longitudinal design. This approach would facilitate a more profound understanding of the relationship between PIU and mental health of older teachers, allowing for a clearer delineation of the direction and strength of the relationships under investigation. Second, while the sample includes teachers from various regions in China, it may not be fully representative of the entire population of older teachers nationwide. The convenience sampling approach, facilitated by the cooperation of local Education Bureaus, was utilized to recruit participants, which could potentially limit the generalizability of our findings. Future studies should recruit larger and more diverse samples from a wider range of regions to enhance the generalizability of the findings and further explore potential regional differences. Third, the study relies on self-report measures, which may be subject to response biases such as social desirability or recall bias. Participants may have underreported their levels of PSU or psychological distress due to social stigma or a lack of awareness of their own behaviors and experiences.

## Data Availability

The raw data supporting the conclusions of this article will be made available by the authors without undue reservation.
