# Supplementary material for: The relationship between problematic internet use and psychological distress in older Chinese teachers (40+) during different stages of the COVID-19 pandemic: three cross-sectional studies
Source: Front Public Health. 2024 Dec 20;12:1442852. doi: 10.3389/fpubh.2024.1442852 (PMC11695432; doi:10.3389/fpubh.2024.1442852)
Supplement: Supplementary file 1 [file Table_1.docx]

**Table S1.** Key characteristics for the participants of Study 1 (*n*=3,929).

| Age in year; n (%) |  |
| --- | --- |
| 40-45 years | 1065 (27.1) |
| 45-48 years | 2864 (72.9) |
| Sex; n (%) |  |
| Male | 1167 (29.7) |
| Female | 2762 (70.3) |
| Type of school; n (%) |  |
| Primary | 2273 (57.9) |
| Secondary | 1656 (42.1) |
| Mentor; n (%) |  |
| Yes | 2288 (58.2) |
| No | 1641 (41.8) |
| Teaching subject; n (%) |  |
| Chinese | 1512 (38.5) |
| Mathematics | 1345 (34.2) |
| English | 725 (18.5) |
| Science (including physics, chemistry, etc.) | 243 (6.2) |
| Others (including PE, music, etc.) | 104 (2.6) |

**Table S2** The items of constructs in current study

| **Constructs** | **Scale** | **Items** |
| --- | --- | --- |
| Fear of COVID-19 | Fear of COVID-19 Scale | 1. 我特别害怕新冠肺炎. I am most afraid of coronavirus-19. |
|  |  | 2. 当我想到新冠肺炎时会很不舒服. It makes me uncomfortable to think about coronavirus-19. |
|  |  | 3. 当我想到新冠肺炎时双手会发冷、发汗. My hands become clammy when I think about coronavirus-19. |
|  |  | 4. 我担心会因为新冠肺炎而失去性命. I worry that I lose my life because of coronavirus-19. |
|  |  | 5. 当我看到新冠肺炎在社交媒体上的相关新闻时，我会很紧张和焦虑. When watching news and stories about coronavirus-19 on social media, I become nervous or anxious. |
|  |  | 6. 因为我担心感染新冠肺炎，我没办法睡好. I cannot sleep because I’m worrying about getting coronavirus-19. |
|  |  | 7. 当我想到会感染新冠肺炎时，我会心跳加快. My heart races or palpitates when I think about getting coronavirus-19. |
| Problematic internet use | Smartphone Application-Based Addiction Scale | 1. 我的智能手机是我生活中最重要的东西. My smartphone is the most important thing in my life. |
|  |  | 2. 我曾因为智能手机的使用，而和我家人或朋友发生冲突。Conflicts have arisen between me and my family (or friends) because of my smartphone use. |
|  |  | 3. 专注使用智能手机是改变我心情的一个途径。Preoccupying myself with my smartphone is a way of changing my mood. |
|  |  | 4. 从开始玩手机起，我玩智能手机的时间越来越长。Over time, I fiddle around more and more with my smartphone. |
|  |  | 5. 当我想玩智能手机却拿不到或玩不成时，我会感到想发脾气，生气、难受或心烦. If I cannot use my smartphone when I feel like, I feel sad, moody, or irritable. |
|  |  | 6. 若我尝试减少使用智能手机的时间，我只能坚持很短一段时间，最终只会跟以前一样，甚至比当初使用得更多。If I try to cut the time I use my smartphone, I manage to do so for a while, but then I end up using it as much or more than before. |
| Psychological distress | 21-item Depression, Anxiety, and Stress Scale | 1. 我觉得很难让自己安静下来. I found it hard to wind down. |
|  |  | 2. 我感到口干. I was aware of dryness of my mouth. |
|  |  | 3. 我好像不能再有任何愉快、舒畅的感觉. I couldn't seem to experience any positive feeling at all. |
|  |  | 4. 我感到呼吸困难（例如不是做运动时也感到气促或透不过气来）. I experienced breathing difficulty (e.g., excessively rapid breathing, breathlessness in the absence of physical exertion). |
|  |  | 5. 我感到很难自动去开始工作. I found it difficult to work up the initiative to do things. |
|  |  | 6. 我对事情往往作出过敏反应. I tended to overreact to situations. |
|  |  | 7. 我感到颤抖（例如手震）. I experienced trembling (e.g. in the hands). |
|  |  | 8. 我觉得自己消耗很多精神. I felt that I was using a lot of nervous energy. |
|  |  | 9. 我忧虑一些令自己恐慌或出丑的场合. I was worried about situations in which I might panic and make a fool of myself. |
|  |  | 10. 我觉得自己对将来没有什么可盼望. I felt that I had nothing to look forward to. |
|  |  | 11. 我感到忐忑不安. I found myself getting agitated. |
|  |  | 12. 我感到很难放松自己. I found it difficult to relax. |
|  |  | 13. 我感到忧郁沮丧. I felt down-hearted and blue. |
|  |  | 14. 我无法容忍任何阻碍我继续工作的事情. I was intolerant of anything that kept me from getting on with what I was doing. |
|  |  | 15. 我感到快要恐慌了. I felt I was close to panic. |
|  |  | 16. 我对任何事也不能热衷. I was unable to become enthusiastic about anything. |
|  |  | 17. 我觉得自己不怎么配做人. I felt l wasn't worth much as a person. |
|  |  | 18. 我发觉自己很容易被触怒. I felt that l was rather touchy. |
|  |  | 19. 我察觉自己在没有明显的体力劳动时，也感到心律不正常. I was aware of the action of my heart in the absence of physical exertion (e.g., sense of heart rate increase, heart missing a beat) |
|  |  | 20. 我无缘无故地感到害怕. l felt scared without any good reason. |
|  |  | 21. 我感到生命毫无意义. I felt that life was meaningless. |
| Psychological Need Thwarting of Online Teaching | Psychological Need Thwarting Scale of Online Teaching | 1. 疫情期间的线上课程进行时，我无法自己决定想要的教学方式。In online courses during the pandemic, I cannot decide for myself how I want to teach. |
|  |  | 2. 疫情期间的线上教学工作进行时，我觉得有股压力会影响我的行为举止，使其符合特定规范In online teaching work during the pandemic, I feel there is pressure that affects my behavior and requires me to comply in a certain way. |
|  |  | 3. 疫情期间我必需遵循某种规定的线上教学方式。I have to follow a prescribed online teaching style during the pandemic. |
|  |  | 4. 疫情期间，我感受到外在环境的压力，限制我必须选定特定的线上教学方式。During the pandemic, I feel pressure from the external environment that limited me in choosing a particular online teaching style. |
|  |  | 5. 疫情期间的工作环境中，有些线上教学的情况让我觉得无能为力。There are some online teaching situations that make me feel incapable in may daily work environment during the pandemic. |
|  |  | 6.我有时会跟人提到疫情期间线上教学工作中让我感到无能为力的事情。I sometimes talk about the things that make me feel powerless to do my online teaching job during the pandemic. |
|  |  | 7. 疫情期间的线上教学工作有时会让我产生无力感。Online teaching during the pandemic sometimes makes me feel powerless. |
|  |  | 1. 由于环境中缺乏磨练机会，我觉得自己不能胜任线上教学的工作任务。Due to the lack of training opportunities in may environment, I feel that I am capable of performing online teaching tasks. |
|  |  | 9. 疫情期间进行线上教学时，我觉得自己与其他同事及领导之间有所隔阂。I feel disconnected from other colleagues and leaders when teaching online during the pandemic. |
|  |  | 10. 疫情期间进行线上教学时，我无法感受到同事及领导对我的关心。I do not feel that my colleagues and leaders care about me when teaching online during the pandemic. |
|  |  | 11. 疫情期间的线上教学取得良好成效时，我觉得同事与领导会嫉妒我。I feel that my colleagues and leaders are jealous of me when I achieve good results in online teaching during the pandemic. |
|  |  | 12. 疫情期间进行线上教学时，我觉得同事与领导不喜欢我。I feel that my colleagues and leaders do not like me when I conduct online teaching during the pandemic. |
| Burnout | Emotional Exhaustion Subscale of the Primary and Secondary School Teachers’ Job Burnout Questionnaire | 1. 我觉得自己在透支生命。 I feel emotionally drained from my work. |
|  |  | 2. 我觉得做老师是一份令人心力交瘁的工作。I feel teaching is really an exhausting job for me. |
|  |  | 3. 工作一天后，我感到筋疲力尽。After a day at work，I feel exhausted. |
|  |  | 4. 我觉得教学工作耗尽了我的情绪和情感。I feel that teaching has exhausted me emotionally and mentally. |
|  |  | 5. 从早到晚，我的脑袋里都有一根弦紧崩着，让我觉得很难受。I feel like I’m at the end of my rope. |
|  |  | 6. 早上起床后我会觉得很疲乏。I feel fatigued when I get up in the morning. |
|  |  | 7. 在工作中我有一种被掏空的感觉。I feel frustrated by my job. |
|  |  | 8. 我觉得我在工作中付出太多。I feel I’m working too hard on my job. |

**Table S3.** Means, Standard Deviation and Pearson correlation among the variables in Study 1 (*n*=3,929).

|  | M (SD) | Prevalence (n, %) | 1 | 2 | 3 |
| --- | --- | --- | --- | --- | --- |
| 1. Fear of Covid-19 | 15.04 (4.20) | Normal: 2802 (71.3%) | 1 |  |  |
|  |  | Abnormal: 1127 (28.7%) |  |  |  |
| 2. PIU | 16.57 (6.29) | Normal: 2854 (72.6%) | 0.36** | 1 |  |
|  |  | Abnormal: 1075 (27.4%) |  |  |  |
| 3. Psychological distress | 7.42 (9.86) | Normal: 3820 (97.2%) | 0.45** | 0.35** | 1 |
|  |  | Abnormal: 109 (2.8%) |  |  |  |

***p* <0.01. PIU, problematic internet use.

**Table S4.** Key characteristics for the participants of Study 2 (*n*=3,502).

| Age in year; n (%) |  |
| --- | --- |
| 40-45 years | 1497 (42.7) |
| 46-50 years | 925 (26.4) |
| 51-55 years | 861 (24.6) |
| 56-60 years | 219 (6.3) |
| Sex; n (%) |  |
| Male | 1755 (50.1) |
| Female | 1747 (49.9) |
| Type of school; n (%) |  |
| Primary | 1657 (47.3) |
| Secondary | 1845 (52.7) |
| Mentor; n (%) |  |
| Yes | 1607 (45.9) |
| No | 1895 (54.1) |
| Teaching subject; n(%) |  |
| Chinese | 996 (28.4) |
| Mathematics | 1144 (32.7) |
| English | 354 (10.1) |
| Science (including physics, chemistry, etc.) | 360 (10.3) |
| Others (including PE, music, etc.) | 648 (18.5) |

**Table S5**. Means, Standard deviation and Pearson correlation among the variables of Study 2 (*n*=3,502).

|  | M (SD) | Prevalence (n, %) |  | 1 | 2 | 3 |
| --- | --- | --- | --- | --- | --- | --- |
| 1. PIU | 17.07 (5.87) | Normal：2544 (72.6%) |  | 1 |  |  |
|  |  | Abnormal：958 (27.4%) |  |  |  |  |
| 2. PNT_Online Teaching | 42.78 (11.08) |  |  | 0.28** | 1 |  |
| 3. Psychological distress | 10.41 (10.39) | Normal: 3367 (96.1%) |  | 0.33** | 0.35** | 1 |
|  |  | Abnormal: 135 (3.9%) |  |  |  |  |

***p* <0.01. PIU, problematic internet use. PNT, psychological need thwarting.

**Table S6.** Key characteristics for the participants of Study 3 (*n*=1,276).

| Age in year; n (%) |  |
| --- | --- |
| 40-45 years | 255 (44.7) |
| 46-50 years | 353 (27.7) |
| 51-55 years | 286 (22.4) |
| 56-60 years | 67 (5.2) |
| Sex; n (%) |  |
| Male | 631 (49.5) |
| Female | 645 (50.5) |
| Type of school; n (%) |  |
| Primary | 716 (56.1) |
| Secondary | 560 (43.9) |
| Mentor; n (%) |  |
| Yes | 577 (45.2) |
| No | 699 (54.9) |
| Teaching subject; n(%) |  |
| Chinese | 382 (29.9) |
| Mathematics | 438 (34.3) |
| English | 117 (9.2) |
| Science (including physics, chemistry, etc.) | 97 (7.6) |
| Others (including PE, music, etc.) | 242 (19.0) |

**Table S7**. Means, Standard deviation and Pearson correlation among the variables in Study 3 (*n*=1,276).

|  | M (SD) | Prevalence (n, %) |  | 1 | 2 | 3 |
| --- | --- | --- | --- | --- | --- | --- |
| 1. PIU | 16.33 (6.20) | Normal：964 (75.5%) |  | 1 |  |  |
|  |  | Abnormal：312 (24.5%) |  |  |  |  |
| 2. Burnout | 27.81 (11.99) |  |  | 0.49** | 1 |  |
| 3. Psychological distress | 10.99 (11.94) | Normal: 1205 (94.4%) |  | 0.43** | 0.51** | 1 |
|  |  | Abnormal: 71 (5.6%) |  |  |  |  |

***p* <0.01. PIU, problematic internet use.
